# Association between number of confidants and adolescent anxiety/depression: a school-based study

**DOI:** 10.1186/s13034-024-00778-0

**Published:** 2024-07-18

**Authors:** Asuka Nishida, Jerome Clifford Foo, Satoshi Yamaguchi, Fumiharu Togo, Shinji Shimodera, Atsushi Nishida, Yuji Okazaki, Tsukasa Sasaki

**Affiliations:** 1 Department of Physical and Health Education, Graduate School of Education, The University of Tokyo, 7-3-1 Hongo, Bunkyo-ku, Tokyo, 113-0033 Japan; 2 Japan Society for the Promotion of Science, Kojimachi Business Center Building, 5-3-1 Kojimachi, Chiyoda-ku, Tokyo, 102-0083 Japan; 3 Department of Genetic Epidemiology in Psychiatry, Central Institute of Mental Health, Medical Faculty Mannheim, University of Heidelberg, J5, 68159 Mannheim, Germany; 4 Department of Psychiatry, College of Health Sciences, University of Alberta, 11315 - 87 Ave NW, AB T6G 2H5 Edmonton, Canada; 5 Neuroscience and Mental Health Institute, University of Alberta, 87 Avenue & 112 Street, AB T6G 2E1 Edmonton, Canada; 6 The Unit for Mental Health Promotion, Research Center for Social Science & Medicine, Tokyo Metropolitan Institute of Medical Science, 2-1-6 Kamikitazawa, Setagaya-ku, Tokyo, 156-8506 Japan; 7 Department of Neuropsychiatry, Seiwa Hospital, 1777 Otu Sakawa, Takaoka, Kochi 789-1202 Japan; 8 Tokyo Metropolitan Matsuzawa Hospital, 2-1-1 Kamikitazawa, Setagaya-ku, Tokyo, 156-0057 Japan

**Keywords:** Adolescents, Anxiety symptoms, Confidants, Depressive symptoms

## Abstract

**Background:**

Having no or few confidants is found to be associated with more severe mental health problems and a higher prevalence of depression in adults, but research examining this association in adolescents is scarce. Social relationships may be particularly critical during adolescence, as it is an important developmental period during which vulnerability to mental health problems increases. The present study examined the relationship between having no or few confidants and anxiety/depressive symptoms in adolescents.

**Methods:**

Cross-sectional self-report survey targeting 7–12th grade students (age range: 12–18) was conducted in public junior and senior high schools in Mie and Kochi, Japan. Data from 17,829 students (49.7% boys) were analyzed. Associations between anxiety/depressive symptoms (12-item General Health Questionnaire; score range: 0–12) and the number of confidants (None, 1–3, or ≥ 4) were examined using multilevel regression analyses. The analyses were stratified by gender and school level (junior/senior high), and adjusted for experiences of being physically abused and bullied and the interactions of these experiences with the number of confidants.

**Results:**

Having no or 1–3 confidants was associated with more anxiety/depressive symptoms, compared to having ≥ 4 confidants (*p* < 0.001) in all stratified groups. Having no confidants was associated with more anxiety/depressive symptoms than having 1–3 confidants (*p* < 0.001); in senior high boys, no difference was observed between having no confidants and having 1–3 confidants. In addition, in senior high boys, victims of bullying who have confidants reported significantly less anxiety/depressive symptoms than the victims who have no confidants (*p* < 0.01).

**Conclusions:**

Adolescents who had no or few confidants had more anxiety/depressive symptoms. Attention needs to be paid to better identify these adolescents, and avenues to support them need to be established.

## Background

Adolescence is an important developmental period when vulnerability to mental health problems increases [6, 23, 24]. Research indicates that 10–20% of children and adolescents worldwide experience mental health problems [25], a number which is increasing [7, 36]. It is shown that these problems are related to adverse outcomes including poor school performance and suicidal ideation [14, 18], and can have negative consequences on later life outcomes, including leading to the development of mental disorders [22].

One of the known risk factors for mental health problems is social isolation and a lack of confidants [4, 46]. Confidants give people someone to talk to about their problems [13, 16], and provide emotional support, having significant protective effects against mental health problems [40, 42]. Data suggest that the negative effects of social isolation on mental health are particularly pronounced during adolescence [6, 35]; peer rejection, bullying, and loneliness are identified as risk factors for depression in adolescents [28, 35]. This may be because adolescents are at a unique period in which they experience profound social transformations and acquire more sophistication in social interactions, with peer and family interactions becoming increasingly important [26, 35].

In healthy adults, it has been reported that having no or few confidants is related to more depressive and anxiety symptoms [8, 16, 38, 44]. One of these studies also implied that depressive symptoms in those having a few confidants may not differ from the depressive symptoms in those having no confidants [44]. In adults suffering from depressive disorders, associations between having no/few confidants and severity of mental health problems have been reported [10, 27, 46, 47]. However, in adolescents, only one study has examined the effects of having no confidants on their mental health problems, finding that adolescents who have no confidants have more depressive symptoms than those with one or more confidants [19].

Various factors may influence the development of depressive symptoms in adolescents. It is observed that girls are more often affected by depression than boys and that the greatest gender difference in depression occurs during adolescence [41]. The adverse effects of having no confidants may also change depending on the different stages of adolescence, given the increasing importance of peer and family interactions in late adolescence [26, 35]. In addition, experiences of being physically abused and bullied are well-established as risk factors for depressive symptoms in adolescents [21, 29] and being a victim of these may also impact the number of confidants, affecting adolescent social relationships at home and in school [5, 31, 48].

Despite adolescents being the most vulnerable group for mental health problems and social isolation, adolescent research on the relationship between the number of confidants and anxiety/depressive symptoms is scarce. Previous work in adolescents has not examined the differences in the effects of having confidants on mental health by factors that are known to influence depression in adolescents [19], such as being physically abused and bullied.

The present study examines the relationship between the number of confidants and anxiety/depressive symptoms in a large cohort of junior and senior high school students. Analyses were stratified by gender and school level (i.e., junior/senior high), and controlled for sociodemographic factors including experiences of being a victim of physical abuse by an adult in the home, and bullying. Since having confidants can potentially counteract the adverse effects of stressful life events and ongoing difficulties [11], the analysis also examined moderating effects of physical abuse/bullying.

## Methods

### Participants and procedures

A cross-sectional survey targeting 7–12th grade students (age range: 12–18) was conducted from 2008 to 2009 in public junior and senior high schools in Japan, using a self-report questionnaire. The principal investigators approached all school principals of public junior and senior high schools in Kochi prefecture (a rural prefecture with some suburban and urban areas clustered around its capital city, with an approximate population of 780,000 at the time of investigation) and in the city of Tsu (the capital city in Mie, a rural prefecture, with an approximate population of 290,000 at the time of investigation), and asked that their schools participate in the survey. The school principals were told that school participation was voluntary.

The parents and guardians received a letter from the principal investigators that asked them to notify the school if they did not wish their child to participate in the survey. On the survey days, the survey questionnaire and an envelope (in which to seal the completed questionnaire before handing in to the teachers) were handed to the students by their teachers at school. The students were told that participation in the study was anonymous and voluntary, and that their answers would be kept confidential. On the survey, students were instructed that they could leave the questions blank if they did not wish to answer. Each teacher reported the total number of students and present and absent on the day of the survey. Of all school schools in Kochi prefecture and the city of Tsu in Mie prefecture (138 junior and 36 senior high schools), 48 and 29 schools participated, respectively. Overall, 19,436 students in the participating schools were asked to participate in the survey. Of these, 798 (4.1%) were absent on the days of the survey, 388 (2.0%) declined to participate, and 146 (0.7%) did not return their questionnaires; a total of 18,104 students (93.1%) participated in the survey.

All methods were carried out in accordance with relevant guidelines and regulations and also adhered with Japan’s Ethical Guidelines for Epidemiological Research. This study and the data collection was approved by the ethics committees of the Tokyo Metropolitan Institute of Psychiatry (approval number: 20-9), the Mie University School of Medicine (approval number: 603), and Kochi Medical School at Kochi University (approval number: 20-57).

### Measures

The questionnaire consisted of three parts, including (1) anxiety/depressive symptoms, (2) the number of confidants, and (3) physical abuse or bullying.

### Anxiety/depressive symptoms

Mental health problems within the past month were assessed using the Japanese version of the 12-item General Health Questionnaire (GHQ-12) [15]. The GHQ-12 is a self-report screening tool for non-psychotic psychiatric symptoms, particularly anxiety and depressive symptoms [15]. The validity of GHQ-12 in adolescents has been previously confirmed [43]. The validity and reliability of the Japanese version of GHQ-12 has been established in people of all ages [30]. Each item of the GHQ-12 was rated on a four-point Likert scale including “less than usual”, “no more than usual”, “fairly more than usual”, and “much more than usual”; the present study applied a bimodal scoring method (0–0–1–1), which creates a possible score range of 0–12. The Cronbach’s alpha using this method was 0.84 in the present sample.

### Number of confidants

The number of confidants was assessed to measure the lack of quantity of social contacts for support, with the following single-item question, “How many confidants do you have, with whom to consult about your worries or troubles?”, with five possible responses including “none,” “one,” “two,” “three,” and “four or more.” For analysis, these responses were merged into three groups: (1) no, (2) a few (1–3), and (3) more (≥ 4) confidants, using cut-off numbers, determined based on combined criteria from previous studies that looked at presence/absence of confidants [19] and into four groups, including none, 1–3, 4–6 and ≥ 7 [44].

### Experiences of being physically abused and bullied

Being a victim of physical abuse/bullying was measured using a single-item question for each type of experience. Being a victim of physical abuse was assessed using the following single yes/no question: “Within the past month, have you experienced physical abuse from adults you live with?” The variable was coded as 1 if the students responded ‘yes’, and 0 if responded ‘no’. Being a victim of bullying was assessed using the following single yes/no question: “Have you been bullied within the past year?” The variable was coded as 1 if the students responded ‘yes’, and 0 if they responded ‘no’.

### Statistical analysis

All statistical analyses were conducted using R version 4.2.1. Listwise deletion was used for participants with missing data. Of the 18,104 students, data from 267 students were missing for the number of confidants. Data from 8 students were excluded for being outside the age ranges of 12–15 for junior high school (JH) and 15–18 for senior high school (SH). Responses from 17,829 students (49.7% boys) were included in the final analyses.

### Descriptive statistics

Differences in anxiety/depressive symptom scores by gender (male/female) and school level (JH/SH) were tested using Welch’s two-sample *t*-test. Differences in the proportion of having no confidants by gender and school level were tested using a chi-square test. Moreover, differences in anxiety/depressive symptom scores by experiences of being physically abused and bullied were tested using Welch’s two-sample *t*-test.

### Association between anxiety/depressive symptom scores and number of confidants

The association between anxiety/depressive symptom scores and the number of confidants was tested using analysis of covariance (ANCOVA), within a multi-level framework considering the school-clustered structure of the data (i.e., students are clustered in schools). ANCOVA was followed by a post-hoc analysis to examine the differences of GHQ-12 scores between groups with no, a few (1–3), and more (≥ 4) confidants, using Tukey–Kramer test (implemented in the ‘emmeans’ package, version 1.7.3). The level of significance was set at alpha = 0.05 in all analyses.

The association was investigated using two models: the first model (Model 1) adjusted for age; the second model (Model 2) adjusted for experiences of being physically abused and bullied in addition to age. Model 2 also included two terms which examined the interactions between (1) physical abuse and the number of confidants, and (2) bullying and the number of confidants. The same models were also calculated stratified by gender and school level.

## Results

### Descriptive statistics

Sample characteristics are shown in Table 1. GHQ-12 scores differed significantly by gender, being higher in girls than in boys (*t*(17,713) = 31.5, *p* < 0.001) and school level, being higher in SH students than in JH students (*t*(17,783) = 16.9, *p* < 0.001).

**Table 1:** Descriptive statistics for GHQ-12 scores, the number of confidants, and covariates (experiences of being physically abused and bullied) in Japanese junior and senior high boys and girls (*N* = 17,829)

	Junior high boys		Senior high boys		Junior high girls		Senior high girls	
	*n* = 4359		*n* = 4491		*n* = 4121		*n* = 4858	
	Mean	SD	Mean	SD	Mean	SD	Mean	SD
Age (years)	13.71	0.91	16.60	0.93	13.73	0.91	16.60	0.92
GHQ-12 Score	2.48	2.77	3.17	3.00	3.85	3.17	4.64	3.19

	*n*	%	*n*	%	*n*	%	*n*	%
Having no confidants	981	22.5	1084	24.1	461	11.2	586	12.1
Having1–3 confidants	1434	32.9	1646	36.6	1894	46.0	2366	48.7
Having ≥ 4 confidants	1944	44.6	1761	39.2	1766	42.8	1906	39.2
Victim of physical abuse	258	5.9	71	1.6	232	5.6	122	2.5
Victim of bullying	528	12.1	212	4.7	438	10.6	188	3.9

Score ranges are 0–12 for GHQ-12 score, 0/1 for victim of physical abuse, and 0/1 for victim of bullying

GHQ-12, 12-item General Health Questionnaire; SD, standard deviation

**Fig. 1:**
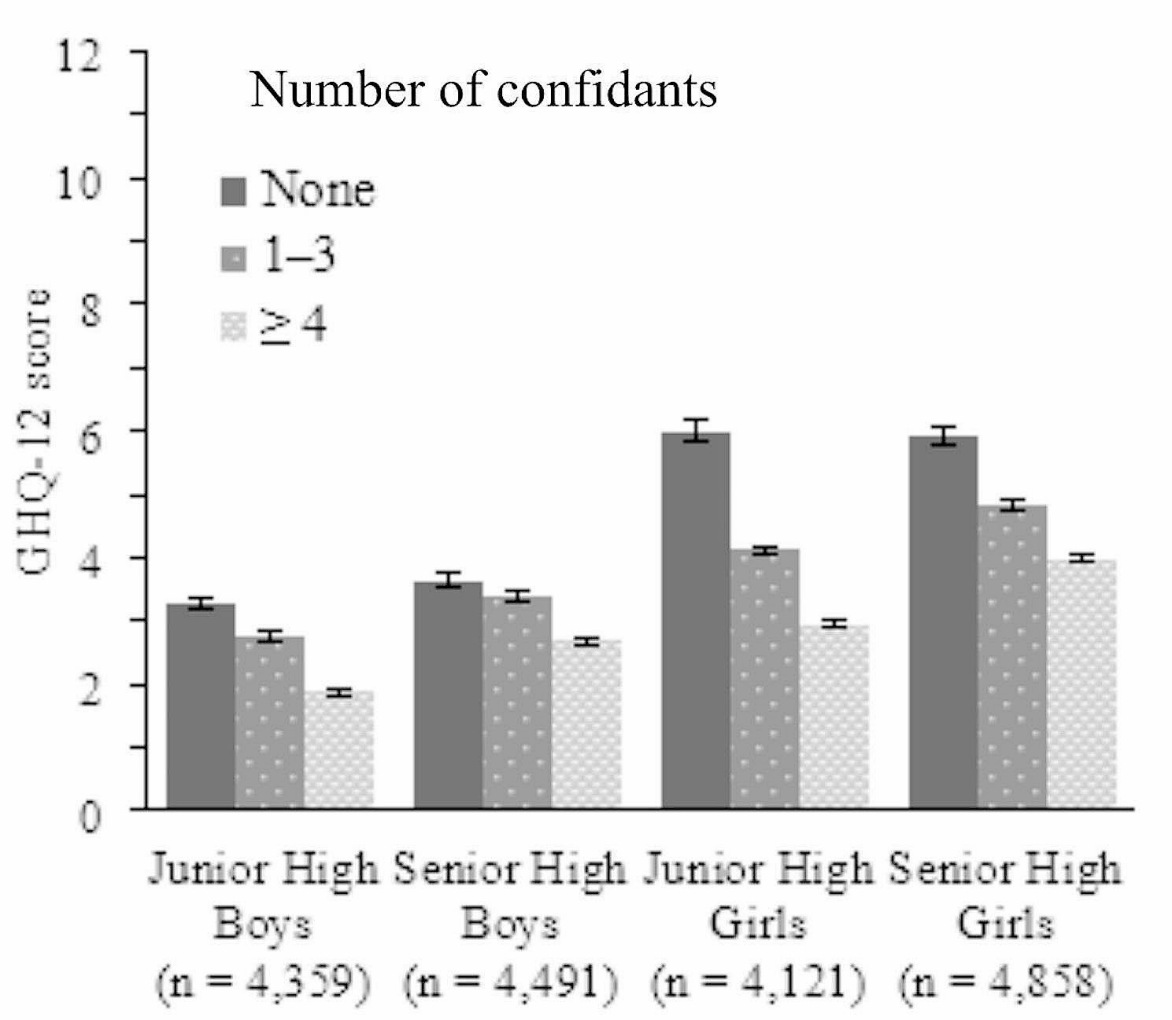
Mean GHQ-12 scores according to the number of confidants. *Note* Error bars show standard error of the mean. GHQ-12, 12-item General Health Questionnaire (score range 0–12)

A significantly greater proportion of boys had no confidants than girls (JH: *X*^2^(1) = 191.2, SH: *X*^2^(1) = 230.9, both *p* < 0.001), but the proportion of having no confidants did not differ by school level (boys: *X*^2^(1) = 3.4, *p* = 0.07, girls: *X*^2^(1) = 1.7, *p* = 0.20). Mean GHQ-12 scores according to the number of confidants are shown in Fig. 1.

Differences in GHQ-12 scores between the victims and non-victims of physical abuse and bullying are shown in Table 2. In all stratified groups, GHQ-12 scores were significantly higher in the victims of physical abuse than non-victims (all *p* < 0.001). GHQ-12 scores were also significantly higher in the victims of bullying than non-victims, in all stratified groups (all *p* < 0.001).

**Table 2:** Comparisons of mean GHQ-12 scores between the victims and non-victims of physical abuse and bullying

	Non-victims	Victims	T
	Mean (SEM)	Mean (SEM)	
Junior high boys			
Physical abuse	2.36 (0.04)	4.36 (0.21)	− 9.39***
Bullying	2.18 (0.04)	4.66 (0.14)	− 16.83***
Senior high boys			
Physical abuse	3.12 (0.04)	6.27 (0.44)	− 7.17***
Bullying	3.03 (0.04)	6.03 (0.24)	− 12.12***
Junior high girls			
Physical abuse	3.71 (0.05)	5.91 (0.21)	− 9.97***
Bullying	3.55 (0.05)	6.17 (0.15)	− 16.21***
Senior high girls			
Physical abuse	4.59 (0.05)	6.45 (0.29)	− 6.40***
Bullying	4.53 (0.05)	7.07 (0.23)	− 10.67***

Values derive from Welch’s two-sample *t*-test

GHQ-12, 12-item General Health Questionnaire (score range 0–12); SEM, standard error of the mean

***p < 0.001

### Association between anxiety/depressive symptom scores and the number of confidants

Effects of the number of confidants, and its interactions with physical abuse/bullying, on GHQ-12 scores is shown in Table 3. The number of confidants was significantly associated with GHQ-12 scores in both Models 1 and 2, as well as in all stratified groups (all *p* < 0.001), except in Model 2 in SH boys. In SH boys, being a victim of bullying was significantly associated with less anxiety/depressive symptoms in those with confidants than those with no confidants (F(*df*s) = 4.75 (2, 4,469), *p* < 0.01). In the rest of the stratified groups, no significant interaction effects between physical abuse/bullying and the number of confidants were observed.

**Table 3:** Effects of the number of confidants on GHQ-12 score before and after adjusting for covariates

	Junior high boys		Senior high boys		Junior high girls		Senior high girls	
	Model 1	Model 2	Model 1	Model 2	Model 1	Model 2	Model 1	Model 2
	F (df)	F (df)	F (df)	F (df)	F (df)	F (df)	F (df)	F (df)
Number of confidants	96.77*** (2, 4355)	107.36*** (2, 4330)	40.48*** (2, 4487)	43.40*** (2, 4469)	202.44*** (2, 4117)	217.01*** (2, 4071)	98.02*** (2, 4854)	98.20*** (2, 4829)
Age	13.17*** (1, 4355)	14.55*** (1, 4330)	0.84 (1, 4487)	0.90 (1, 4469)	20.02*** (1, 4117)	21.48*** (1, 4071)	0.06 (1, 4854)	0.09 (1, 4829)
Experience of being physically abused	–	135.42*** (1, 4330)	–	75.16*** (1, 4469)	–	100.75*** (1, 4071)	–	34.29*** (1, 4829)
Experience of being bullied	–	334.53*** (1, 4330)	–	166.17*** (1, 4473)	–	228.23*** (1, 4071)	–	100.59*** (1, 4829)
Number of confidants * Experience of being physically abused	–	0.47 (2, 4330)	–	1.36 (2, 4469)	–	0.16 (2, 4071)	–	0.485 (2, 4829)
Number of confidants * Experience of being bullied	–	1.81 (2, 4330)	–	4.75** (2, 4469)	–	2.09 (2, 4071)	–	0.50 (2, 4829)

Values derive from ANCOVA with multilevel regression analyses. Score ranges are 0–12 for GHQ-12 score, 0/1 for the absence/presence of physical abuse, and 0/1 for the absence/presence of bullying. Covariates include experience of being physically abused in the past month, experience of being bullied in the past year, and the interactions between physical abuse/bullying and the number of confidants, in addition to age

df, degrees of freedom; GHQ-12, 12-item General Health Questionnaire

**p < 0.01, ***p < 0.001

Multiple comparisons of estimated mean GHQ-12 scores between groups with no, 1–3, and ≥ 4 confidants are shown in Table 4. Model 1 shows the association before adjusting for experiences of being physically abused and bullied. In JH boys, having 1–3 confidants was significantly associated with lower GHQ-12 scores compared to having no confidants (*p* < 0.001). In both JH and SH boys, having ≥ 4 confidants was also significantly associated with lower GHQ-12 scores compared to having no confidants (*p* < 0.001). Furthermore, in both JH and SH boys, having ≥ 4 confidants was significantly associated with lower GHQ-12 scores compared to having 1–3 confidants (*p* < 0.001). In SH boys, no significant difference in GHQ-12 scores was observed between having 1–3 and no confidants (*p* = 0.12).

**Table 4:** Differences in estimated mean GHQ-12 scores between groups with no, 1–3, and ≥ 4 confidants

	Junior high boys				Senior high boys				Junior high girls				Senior high girls			
	Model 1^a^		Model 2^b^		Model 1^a^		Model 2^b^		Model 1^a^		Model 2^b^		Model 1		Model 2^b^	
	Β	SE	Β	SE	Β	SE	Β	SE	Β	SE	Β	SE	Β	SE	Β	SE
1–3 Confidants (Ref. None)	− 0.52***	0.11	− 0.59*	0.22	− 0.25	0.12	− 0.88	0.42	− 1.92***	0.16	− 1.69***	0.29	− 1.13***	0.14	− 1.37**	0.46
≥ 4 Confidants (Ref. None)	− 1.25***	0.10	− 1.23***	0.22	− 0.96***	0.11	− 1.79**	0.51	− 3.00***	0.16	− 2.79***	0.31	− 1.96***	0.15	− 2.48***	0.49
≥ 4 Confidants (Ref. 1–3)	− 0.73***	0.09	− 0.64**	0.21	− 0.71***	0.10	− 0.92	0.49	− 1.11***	0.10	− 1.10***	0.25	− 0.82***	0.10	− 1.11*	0.39

Values derive from multiple comparisons of the estimated means, with Tukey–Kramer test used for *p-*value adjustments

SE, standard error; GHQ-12, 12-item General Health Questionnaire; Ref, reference group

^a^Adjusted for age

^b^Adjusted for experience of being a victim of physical abuse in the past month, experience of being bullied in the past year, and interactions of these experiences with the number of confidants, in addition to age

*p < 0.05, **p < 0.01, ***p < 0.001

In both JH and SH girls, having 1–3 and ≥ 4 confidants were significantly associated with lower GHQ-12 scores (both *p* < 0.001) compared to having no confidants. Furthermore, in both JH and SH girls, having ≥ 4 confidants was significantly associated with lower GHQ-12 scores compared to having 1–3 confidants (both *p* < 0.001).

The association between having confidants and GHQ-12 scores remained significant after adjusting for covariates (Model 2 in Table 4), except for the comparison between having 1–3 and ≥ 4 confidants in SH boys.

### Analyses adjusted for physical abuse/bullying and its interactions with the number of confidants

In Model 2, physical abuse/bullying were both significantly associated with higher GHQ-12 scores compared to those without these experiences, in all stratified groups (*p* < 0.001 in JH boys and girls and SH boys, and *p* < 0.01 in SH girls; see Table 3).

## Discussion

The present school-based study suggests that having no/few confidants is associated with anxiety/depressive symptoms in adolescents. We showed for the first time that adolescents having no confidants had significantly more anxiety/depressive symptoms than those having a small number (1–3) of confidants, while those having a few confidants had significantly more symptoms than those having 4 or more. These results extend previous adolescent research showing that having no confidants is related to depressive symptoms [19]. Our results suggest that having even a few confidants is a predictor of better mental health compared to having no confidants. Considering that mental health problems in adolescents are not easy to recognize [1, 2, 45], identifying adolescents with no confidants may make it easier to provide support where it is most needed.

The effect of having fewer confidants on higher anxiety/depression scores was larger in girls than in boys, and in JH students than in SH students. The gender difference observed in our study is in line with the findings from previous research showing that the relationship between social support and mental well-being in adolescents is significantly stronger in girls compared to boys [9]. The difference by school level is in line with findings from previous research showing that the relationship between social support from peers and depression is significantly stronger in JH students compared to SH students [40].

Physical abuse and bullying had strong associations with anxiety/depressive symptoms, which is in line with previous research in adolescents showing similar relationships [3, 21, 29]. In addition, the victims of physical abuse/bullying tend to have more difficulty obtaining social support than non-victims [20, 32]. In the present study, having no/few confidants showed a significant effect on anxiety/depressive symptoms, after controlling for physical abuse/bullying and its interaction effects with the number of confidants. This suggests that having no/few confidants can be an indicator of mental health problems in adolescents independently of the presence of physical abuse/bullying.

However, in senior high boys, having few confidants did not show significant association with anxiety/depressive symptoms (Table 4); the reason for this is unclear. Also, the victims of bullying with confidants reported less anxiety/depressive symptoms than the victims of bullying with no confidants in senior high boys (Table 3). These findings are consistent with a previous review on the association between the presence of a confidant and mental health problems which suggested that having a confidant lowers the adverse effects of stressful life events on mental health, rather than a direct relationship between having a confidant and mental health [11]. Our results suggest that having confidants may be a significant protective factor for mental health of the victims of bullying in senior high boys.

In the present study, we found that on the whole, adolescents with no confidants had more anxiety/depressive symptoms. Underlying this was gender differences; on the individual level, girls who had no confidants had poorer mental health than their boy counterparts. However, at the group level, a higher proportion of boys reported no confidants than girls; the percentage of not having confidants in boys was 23.3% and in girls was 11.6% (Table 1). That is, while individual girls might be more severely affected, a larger number of boys may be at risk for mental health problems. It has been suggested that boys have more difficulty recognizing their mental health problems [39] and are more prone to engage in externalizing behavior [17]. The rate of suicide is also higher in boys than in girls [37]. Taken together, our results suggest that girls and boys with no confidants must be given equal attention, although perhaps in different ways.

Furthermore, in the present study, the association between having no confidants and anxiety/depressive symptoms was weaker in SH students than in JH students. One possible explanation is that these groups could differ in the type of relationships with confidants (e.g., parents, family members, peers). From early to middle adolescence, the proportion of those who prefer parents over others as primary confidants is reported to decrease [34], and research has also indicated that adolescents who do not report parents as confidants have worse mental health [12, 33, 40]. Future studies should explore the effects and take into account the nature of confidant relationships.

This study had several limitations. First, the study was cross-sectional and does not allow causal inferences. Second, socioeconomic status (SES) was not included in the present analyses; while data for SES was unavailable for the present study, we note that previous work has reported that the association between the absence of confidants and mental health problems remained significant even after the inclusion of SES [19]. Third, the data was collected in 2008–2009, and whether the same associations would also be obtained from recently collected data is unclear as social relationships via online platforms have changed everyday communication in the past decade, potentially impacting the association between the number of confidants and anxiety/depressive symptoms. Fourth, the participation rate of schools was relatively low especially for junior high school (35% for junior and 80% for senior high schools), and schools which decided to participate in the survey may differ from those which did not. This may reflect higher recognition of the importance of mental health in schools at the senior high level. Fifth, the interpretation of our results should be conservative as we sampled schools from only Kochi prefecture and the city of Tsu in Mie prefecture. However, in general, the school system is similar among all schools in Japan, and our sample would be considered representative of the national sample.

## Conclusions

The present study, conducted in the largest sample to date, suggests that having more confidants is associated with less anxiety/depressive symptoms in adolescents independently of experiences of being physically abused and bullied. It may be possible for family members and educators to recognize those at greater risk of mental health problems and provide more support. Further longitudinal studies are needed to examine directional and dynamic effects of the relationship between the number of confidants and adolescent mental health.

## Data Availability

No datasets were generated or analysed during the current study.
